# The Financial Strength of Our Voluntary Charities

**Published:** 1895-01-26

**Authors:** 


### The Institutional Workshop.

### THE FINANCIAL STRENGTH OF OUR VOLUNTARY CHARITIES.

The following leader is taken from the *Times* of the 23rd instant. It very fairly sums up the discussion, and administers a well-merited rebuke to one person with whom Mr. Burdett declined to enter into any controversy, notwithstanding that he had obtained access to the columns of the *Times*.

It is highly probable that the correspondence which has appeared in our columns upon the question "Is charity *in extremis*?" has caused in many minds a feeling of bewilderment not well suited to stimulate benevolence. By an unfortunate initial mistake, which he would have been thought the last man to commit, Mr. Henry C. Burdett introduced greater confusion into the discussion than he is altogether willing to admit. We must say in passing that this mistake affords no justification whatever for sneers at Mr. Burdett's general attitude, or for any affectation of doubt concerning his motives. For a quarter of a century he has given an immense amount of hard work and of hard cash to the cause of charity; and it is unjust and ungenerous in the extreme to lose sight of that fact because he has been caught tripping. But his mistake, which he was the first to discover and rectify in our columns, affects the general argument to a greater degree than he seems to suppose, and has offered controversial temptations to other people which they have not been able wholly to resist. What he did was to transpose the income and expenditure columns in a table dealing with metropolitan general and special hospitals, and containing the figures prepared and published by the council of the Metropolitan Hospital Sunday Fund. By this transposition he made it appear that the total income of these hospitals for 1893 exceeded that for 1885 by £198,229; and that their total expenditure for 1893 exceeded that for 1885 by only £65,076. A careful reader of the passage which immediately follows the table would suspect that something is wrong with these very roseate figures, and it was possibly the perusal of that passage in print which led Mr. Burdett to re-examine his table. As a matter of fact, the income for 1893 exceeded that for 1885 by only £65,076, while the expenditure for 1893 was in excess of that for 1885 by £198,229. Mr. Burdett says the correction does not affect his contention that the income of the London hospitals is not diminishing. But though this is true technically, it ignores the patent truth that the ability of the hospitals to cope with the work they have to do has diminished. They have felt themselves bound to spend in excess of their expenditure for 1885 three times the sum representing their increase of income. In these matters every useful comparison must take into account the growth of population and of consequent or concomitant demands upon the resources of the hospitals. There is another consequence of Mr. Burdett's mistake to which he does not advert at all. As his figures originally stood, the income of the hospitals for both years appeared to be in excess of their expenditure. As they stand after correction they show that in both years the expenditure was in excess of income. This obviously has an important bearing upon the application of legacies to current expenses, and upon the formation of reserve funds.

Mr. Burdett's corrected figures show that in 1885 the expenditure of the metropolitan general and special hospitals exceeded their income by £40,335, while in 1893 the excess of expenditure over income was £173,488. The increase in the number of out-patients, to take only one item of work, was 367,302, so that the large increase in the deficit cannot be ascribed to lavish conduct of the same volume of business. Now, we are not much concerned about the exact phrase which may be used to qualify this four-fold increase of the deficit. "Charity *in extremis*" may be thought by some too strong, while others will probably accept it as tolerably descriptive of the actual state of affairs. There, however, is the fact that the hospitals spent in 1893 £170,000 in excess of their income, while in 1885 they escaped with a deficit of £40,000. They are enabled to balance their accounts by treating legacies as income, and it would require rather a close investigation of several things which do not appear in this discussion to enable us to decide how far this practice is financially justifiable. Legacies are not a fixed quantity for hospitals in general, or even a steady average quantity; and if we take individual hospitals we shall probably find that their fluctuations are greater than those of the aggregate. Up to a certain point prudence unquestionably prescribes the treatment of occasional windfalls as capital; but after that point is reached we have no difficulty in agreeing with Mr. Burdett that legacies may, and ought to be, dealt with as current income. The real difficulty is to determine the proportions in which an uncertain income may properly be allocated to the two purposes. We gather from Mr. Burdett that there are years in which the ordinary income with all the legacies added does not show a balance on the right side. As the demands upon the hospitals are constantly growing, probably with even greater rapidity than the population, it can hardly be maintained that their position is so unquestionably sound as to make the reversal of Mr. Burdett's columns immaterial to the argument, or to condemn all anxiety on the part of their managers as unjustifiable pessimism.

At the same time we have pleasure in agreeing with Mr. Burdett that nothing is gained by making out things to be worse than they are. The sensational appeals in which some managers indulge repel at least as many people as they attract. If they became universal they might easily engender a mischievous belief in the impossibility of maintaining the voluntary system by any practicable sacrifices. What we want is the plain, unadorned truth, stated as forcibly as any one can state it, but set forth without exaggeration and without unworthy appeals to prejudice. Hospitals, of course, are only a part of the great system of charity in this country, but they are a very important part, and they are more susceptible than others of fairly accurate examination. It may be noted that, taking the country as a whole, they are in a much better position than the hospitals of the metropolis. That is a fact which metropolitan managers would do well to consider with the greatest care. It can hardly be doubted that it is explained, at least in part, by the extraordinary multiplicity of institutions in London. There are, we believe, something like 180 general and special medical charities, of all degrees of importance. One can hardly doubt that this number is far too large, or that a disproportionate share of the total subscriptions is spent upon staff and advertising expenses. Small special hospitals are frequently the result either of misdirected officiousness or of private self-seeking. They cannot fail to be more costly in proportion to the work done than special wards in large general hospitals, because they have to fight much harder for prominence and their general management absorbs a much larger percentage of income. Diffusion of knowledge among benevolent people would do much to effect a diversion of money into the most useful channels, but the process is slow and is constantly thwarted by impulse, without which, indeed, charities of all kinds would languish. The medical profession might perhaps lend valuable assistance both in educating the charitable public and in effecting some organisation of charitable institutions to which by degrees the public would learn to look for authoritative advice.

